# Chronic obstructive pulmonary disease exacerbation in the intensive care unit: clinical, functional and quality of life at discharge and 3 months of follow up

**DOI:** 10.5935/0103-507X.20170008

**Published:** 2017

**Authors:** Renata Cristina Teixeira Pinto Viana, Mariangela Pimentel Pincelli, Emílio Pizzichini, André Pacheco Silva, Joice Manes, Tatiana Dias Marconi, Leila John Marques Steidle

**Affiliations:** 1 Clínica Médica, Universidade do Vale do Itajaí - Itajaí (SC), Brasil.; 2 Terapia Intensiva e Cuidados Paliativos, Universidade Federal de Santa Catarina - Florianópolis (SC), Brasil.; 3 Departamento de Clínica Médica/Pneumologia, Hospital Universitário, Universidade Federal de Santa Catarina - Florianópolis (SC), Brasil.; 4 Terapia Intensiva, Hospital Nereu Ramos - Florianópolis (SC), Brasil.; 5 Instituto de Cardiologia de Santa Catarina - Florianópolis (SC), Brasil.; 6 Clínica Médica, Hospital Universitário, Universidade Federal de Santa Catarina - Florianópolis (SC), Brasil.; 7 Hematologia, Centro de Pesquisas Oncológicas - Florianópolis (SC), Brasil.

**Keywords:** Chronic obstructive pulmonary disease, Pulmonary function tests, Cognition, Quality of life, Treatment outcome

## Abstract

**Objective:**

The purpose of this study was to evaluate the clinical/functional aspects and quality of life of chronic obstructive pulmonary disease patients who were discharged after an intensive care unit admission for acute respiratory failure.

**Methods:**

This prospective study included chronic obstructive pulmonary disease patients who were admitted to two intensive care units between December of 2010 and August of 2011 and evaluated over three visits after discharge. Thirty patients were included, and 20 patients completed the three-month follow up.

**Results:**

There was a significant improvement in the following: forced expiratory flow in one second (L) (1.1/1.4/1.4; p = 0.019), six-minute walk test (m) (- /232.8 /272.6; p = 0.04), BODE score (7.5/5.0/3.8; p = 0.001), cognition measured by the Mini Mental State Examination (21/23.5/23.5; p = 0.008) and quality of life measured by the total Saint George Respiratory Questionnaire score (63.3/56.8/51, p = 0.02). The mean difference in the total score was 12.3 (between visits 1 and three). Important clinical differences were observed for the symptom score (18.8), activities score (5.2) and impact score (14.3). The majority of participants (80%) reported they would be willing to undergo a new intensive care unit admission.

**Conclusion:**

Despite the disease severity, there was a significant clinical, functional and quality of life improvement at the end of the third month. Most patients would be willing to undergo a new intensive care unit admission.

## Abstract (pt)

**Objetivo:**

Avaliar aspectos clínicos e funcionais, assim como qualidade de vida de pacientes com doença pulmonar obstrutiva crônica após receberem alta da unidade de terapia intensiva à qual foram admitidos por insuficiência respiratória aguda.

**Métodos:**

Estudo prospectivo que incluiu pacientes com doença pulmonar obstrutiva crônica admitidos a duas unidades de terapia intensiva entre dezembro de 2010 e agosto de 2011, e que foram avaliados em três visitas após a alta da unidade de terapia intensiva. Incluíram-se 31 pacientes e, destes, 20 pacientes completaram o seguimento de 3 meses.

**Resultados:**

Ocorreu melhora significante dos seguintes aspectos: volume expiratório forçado em 1 segundo (L) (1,1/1,4/1,4; p = 0,019), Teste de Caminhada de 6 Minutos (m) (- /232,8/272,6; p = 0,04), escore BODE (7,5/5,0/3,8; p = 0,001), cognição avaliada com uso da escala *Mini Mental State Examination* (21/23,5/23,5; p = 0,008) e qualidade de vida avaliada pelo *Saint George Respiratory Questionnaire* (63,3/56,8/51; p = 0,02). A diferença média no escore total foi de 12,3 (entre as visitas um e três). Observaram-se diferenças clínicas importantes em relação ao escore de sintomas (18,8), escore de atividades (5,2) e escore de impacto (14,3). A maior parte dos participantes (80%) relatou que aceitaria uma nova admissão à unidade de terapia intensiva.

**Conclusão:**

Apesar da gravidade da doença, ao final do terceiro mês ocorreu uma significativa melhora clínica, funcional e de qualidade de vida. A maior parte dos pacientes aceitaria submeter-se a uma nova internação na unidade de terapia intensiva.

## INTRODUCTION

Chronic obstructive pulmonary disease (COPD) is one of the most common chronic health care problems.^(1,2)^ According to the Global Initiative for Chronic Obstructive Lung Disease (GOLD), COPD is considered a preventable and treatable disease, which is often underdiagnosed in Latin America.^(1)^

The natural course of COPD is characterized by exacerbations, which are considered acute worsening of the respiratory condition, from the stable state and beyond normal day-to-day variations, and these exacerbations require additional treatment. In severe exacerbations, COPD patients require hospitalization or an emergency visit. Both respiratory failure with ventilatory support and intensive care unit (ICU) admission are frequent.^(1-10)^

There are many studies on mortality in severe COPD exacerbations,^(3-15)^ and some studies have analyzed the health status and quality of life of patients with COPD during the stable disease phases. However, there are few studies on the impact of severe exacerbations on the quality of life in this population.^(5,8,16,17)^

Studies that evaluate the impact of an ICU stay in a more comprehensive, detailed form for COPD patients, especially immediately after discharge and in long term follow-up, are necessary.

The present study aims to evaluate patients with COPD during the first three months after they have been discharged from hospitalization for acute respiratory failure (ARF) in two ICUs with consideration for the clinical, functional (pulmonary function/motor function/cognitive function) and quality of life aspects.

## METHODS

A prospective cohort study was conducted between December 2010 and August 2011, involving all COPD patients who were admitted for ARF and discharged from two ICUs in Florianopolis, Santa Catarina, Brazil: 1) *Hospital Universitário* - *Universidade Federal de Santa Catarina* (HU-UFSC) and 2) *Hospital Nereu Ramos* (HNR).

The inclusion criteria were patients over 18 years old; clinical diagnosis of ARF and admitted to the ICU; airflow obstruction of the airways, defined as a ratio of forced expiratory flow in one second (FEV1) to the forced vital capacity (FVC) of less than 0.7; and a history of tobacco smoking. The exclusion criteria were a previous diagnosis of asthma; COPD patients who were hospitalized for reasons other than ARF; and lack of fulfillment of the GOLD criteria.^(1)^

The protocol was approved by the *Universidade Federal de Santa Catarina* Ethics Committee number 1161/10. All participants or relatives gave written, informed consent before entering the study.

During the first week after ICU discharge, the following demographic and ICU admission data were collected: modified Medical Research Council dyspnea scale (mMRC),^(18)^ smoking status, comorbidities, home oxygen therapy before admission, COPD treatment before hospitalization, previous ICU admissions, length of ICU stay, Acute Physiology and Chronic Health Evaluation II (APACHE II) score,^(19)^ and ventilatory support. Three visits were performed as follows: (1) first week after ICU discharge (in patients who were still in the hospital, evaluation was performed at the research hospital), (2) one month after discharge, and (3) three months after discharge.

The demographic data, smoking history, comorbidities, dyspnea score (mMRC), previous COPD treatment features, previous ICU admissions, APACHE II score and ventilatory support history were collected at ICU admission. The following parameters were evaluated in three visits: FVC, FEV_1_, FEV_1_/FVC (spirometric data collected when the maneuver could be performed),^(20)^ body mass index (BMI), six-minute walk test distance (6MWT),^(21)^ mMRC dyspnea score, BODE index score,^(22)^ mini mental (Mini Mental State Examination - MMSE)^(23)^ and Saint George Respiratory Questionnaire (SGRQ) scores.^(24,25)^ Additionally, patients were asked about advanced directives and possible ICU readmission, if necessary.

### Statistical analysis

Statistical analysis was performed using the Statistical Package for Social Science (SPSS) version 17. Descriptive analysis was performed using the frequencies for categorical variables and mean and standard deviation for normal continuous variables. The comparison between the parametric continuous variables was performed using the *t* test and ANOVA with Bonferroni correction and between the categorical variables using chi-square and Fischer's tests. Statistical significance (p) was set as less than or equal to 0.05.

## RESULTS

**Figure 1:**
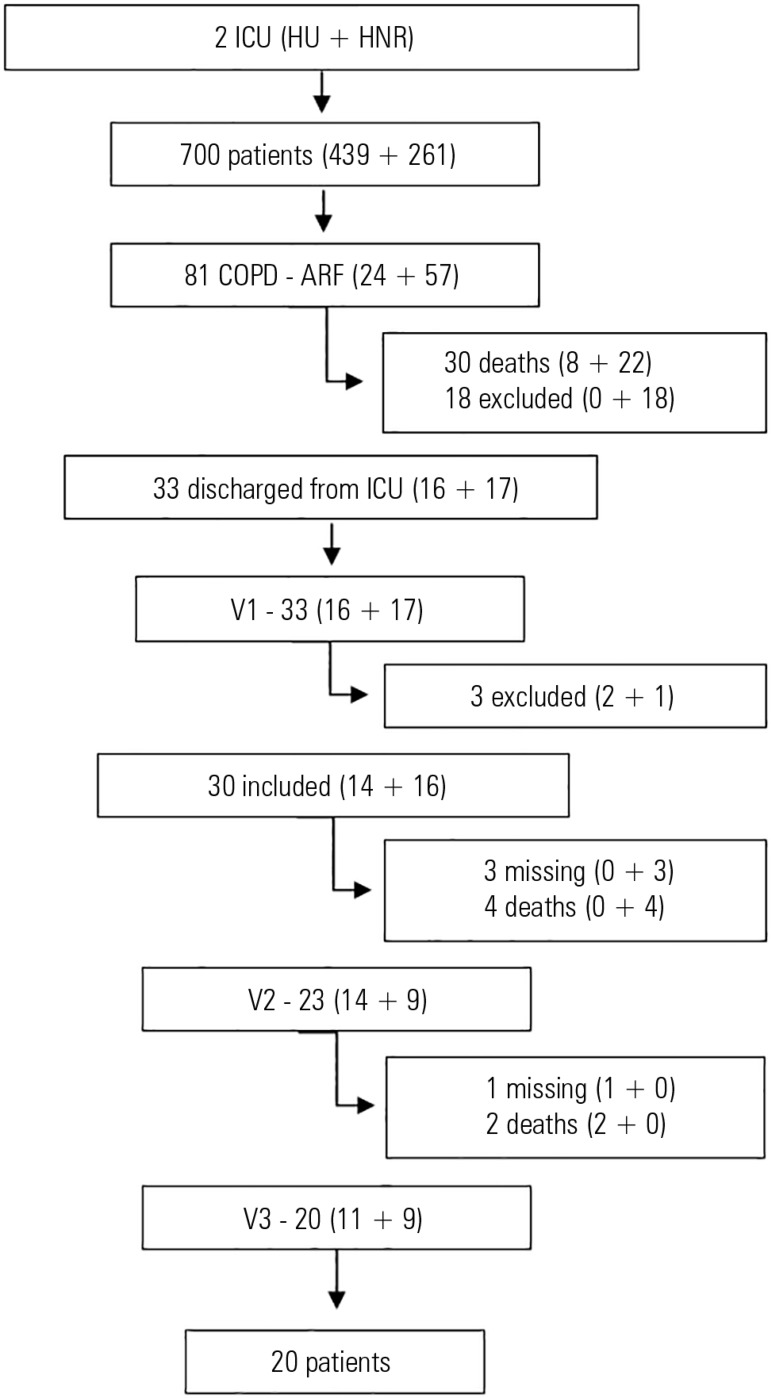
Recruitment and follow up of patients from two intensive care units. ICU - Intensive Care Unit; HU - *Hospital Universitário*; HNR - *Hospital Nereu Ramos*; COPD - chronic obstructive pulmonary disease; ARF - acute respiratory failure; V1 - visit during the first week after discharge from the intensive care unit; V2 - visit one month following discharge from the intensive care unit; V3 - visit three months after discharge from the intensive care unit.

Overall, in the HU-UFSC ICU, 439 patients were admitted to the ICU during the study period; 24 were COPD patients who were hospitalized due to ARF. Eight of these patients died during the ICU stay, and 16 were discharged. Sixteen patients attended the first visit; however, two of them were excluded for not having a FEV_1_/FVC < 0.7. Fourteen patients attended a second visit, but two died before the last visit, and one missed the final follow-up appointment. Eleven patients in this unit completed the last visit (Figure 1).

In the HNR ICU, 261 patients were hospitalized during the study period; 57 of the patients were COPD patients hospitalized due to ARF. During the ICU stay, 22 patients died, and 18 did not meet the clinical criteria necessary for study inclusion. Among the 17 patients who attended the first visit, one was excluded for not having a FEV_1_/FVC < 0.7, three were lost to follow up, and four died before the second visit. In this unit, nine patients completed the study (Figure 1).

After ICU discharge, 30 patients were included in the study. Twenty patients completed the three visits and were included in the analysis (Figure 1).

Considering all 30 patients included in the study, the mean age was 63 ± 8.8 years old, and most patients (70%) were male. Almost half (43.3%) of the patients continued to smoke until their ICU admission, and they had an important smoking history (mean 35 ± 57 packs/year). The mean APACHE II was 21.6 ± 9.6, and the mean ICU stay was 13.5 + 9.6 days. Non-invasive ventilation (NIV) was used in 33% of patients as the first ventilatory support; in 83.3%, invasive support was applied. The demographic data are presented in table 1.

Regarding the referred comorbidities, systemic arterial hypertension (SAH) was present in 58.6%, congestive heart failure (CHF) in 34.5%, diabetes in 27.6% and depression in 34.5% of the cases.

**Table 1:** Characteristics of 30 chronic obstructive pulmonary disease patients discharged from intensive care units

Characteristics	Results
Age (years)	63 (46 - 69)
Males	70
Smoker at ICU admission	43.3
Number of pack-years	57.3 ± 35
2 or more comorbidities	65.5
mMRC before ICU 0/1/2/3/4	10.3/17.2/13.8/13.8/44.8
Domiciliary oxygen therapy at ICU admission	24.1
COPD treatment prior ICU	86.2
Long acting beta agonist	58.6
Long acting anticholinergic bronchodilators	17.2
Previous ICU admissions	27.5
Length of stay in the ICU	13.5 ± 9.6
APACHE II	21.6 ± 8.3
NIV as first ventilatory support	33
Invasive ventilatory support	83.3

ICU - intensive care unit; MMRC - Modified Medical Research Council; COPD - chronic obstructive pulmonary disease; APACHE II - Acute Physiology and Chronic Health Evaluation II; NIV - non-invasive ventilation. The results are expressed as the median (25% - 75%), mean ± standard deviation or percentage.

**Figure 2:**
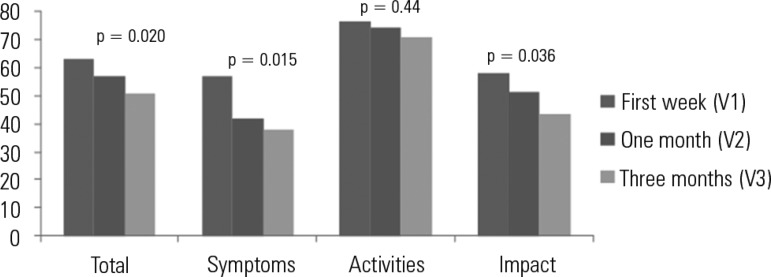
Saint George Respiratory Questionnaire scores in the chronic obstructive pulmonary disease patients during the first week (V1), one month (V2) and three months (V3) after intensive care unit discharge.

As shown in table 2, the follow-up at three months after ICU discharge showed a significant improvement in the quality of life, as perceived by patients, according the total SGRQ score (63.3 *versus* 56.8 *versus* 51, p = 0.02) and most domain scores of this questionnaire (Figure 2). The magnitude of the improvement in the quality of life was also verified through the mean differences scores, and the domain variation values, considering the interval between the first and third visits. The minimal important clinical difference was 4 units.^(26)^ The mean difference in the total score was 12.3. Important clinical differences were observed for the symptoms score (18.8), activities score (5.2) and impact score (14.3) (Table 3).

**Table 2:** Analysis of clinical, functional and quality of life characteristics of 20 patients who completed all three visits after discharge from the intensive care unit

Characteristics	V1	V2	V3	p value
FEV1 post BD (L)	1.1 ± 0.4	1.4 ± 0.5	1.4 ± 0.5	0.019
FEV1 post BD %	40.7 ± 17.4	49.4 ± 15	48.1 ± 15.4	0.120
BMI	27.2 ± 8.8	25.8 ± 8.5	27.5 ± 8.9	0.079
6MWT	---	232.8 ± 128.3	272.6 ± 125.7	0.040
mMRC				V1/2 - 0.700
mMRC 0 a 1	5	30	55	V1/3 - 0.450
mMRC 2 a 4	95	70	45	V2/3 - 0.012
BODE	7.5 ± 1.9	5 ± 2.5	3.8 ± 2.3	0.001
Mini mental	20.9 ± 4.3	23.5 ± 3.8	23.5 ± 3.9	0.008
SGRQ				
Total score	63.3 ± 18.1	56.8 ± 17.5	51 ± 17.1	0.020
Symptoms score	56.7 ± 21.5	41.7 ± 24.2	37.9 ± 23.3	0.015
Activities score	76.3 ± 19.6	74.3 ± 20.6	71.1 ± 17.2	0.444
Impact score	57.9 ± 22	51.4 ± 21.7	43.6 ± 20.3	0.036
Return to ICU, if necessary			80	

V1 - visit during the first week after discharge from the intensive care unit; V2 - visit one month following discharge from the intensive care unit; V3 - visit three months after discharge from the intensive care unit; ICU - intensive care unit; p - significance level; FEV_1_ post BD (L) - forced expiratory volume in 1^st^ second post bronchodilator (in liters); FEV_1_ post BD % - percentage of forced volume in 1^st^ second post bronchodilator; BMI - body mass index; 6MWT - six-minute walk test; MMRC - modified Medical Research Council; BODE - Body-Mass Index, Airflow Obstruction, Dyspnea and Exercise Capacity; SGRQ - Saint George Respiratory Questionnaire. The results are expressed as the mean ± standard deviation or percentage.

**Table 3:** Mean differences in the Saint George Respiratory Questionnaire scores between V1 and V3

Characteristics	≠ V1 - V3
Saint George Respiratory Questionnaire	
Total score	12.3
Symptom score	18.8
Activities score	5.2
Impact score	14.3

V1 - during the first week after intensive care unit discharge; V3 - three months after intensive care unit discharge.

A significant improvement in the FEV_1_ (L) (1.1 *versus* 1.4 *versus* 1.4, p = 0.019) and integrative BODE score (7.5 *versus* 5 *versus* 3.8, p = 0.001) was observed. There was also a significant improvement in the performance on the 6MWT (m) between the second and third visits (232.8 *versus* 272.6, p = 0.040). However, the 6MWT cannot be completed in most patients in the first visit because of their poor clinical conditions. The mMRC dyspnea scale showed significant improvement in the interval between the second and third visits (p = 0.012). The cognitive parameters inferred by examining the Mini Mental showed significant improvement between V1 and V2, and they remained stable between the V2 and the V3 (21 *versus* 23.5 *versus* 23.5, p = 0.008) (Table 2).

The survival rates during the ICU stay, one month after discharge, and three months after discharge were 63%, 59% and 56%, respectively. During the follow-up, six patients (6/30) died (20%). After three months, patients were asked about their willingness to undergo a new admission in intensive care, if necessary. Even while aware of the possibility of negative outcomes, the great majority (80%) said they would agree to another ICU admission (Table 2).

## DISCUSSION

The main finding of the present cohort study was the observation of a significant quality of life improvement detected by the SGRQ within three months after ICU discharge. In accordance with this result, there was also a significant improvement in the FEV_1_, 6MWT distance, BODE index and cognitive function measured by the MMSE. We note that our entire study population of 30 patients had severe dyspnea, and there was also a high frequency of ongoing smokers at ICU admission. Some patients were already undergoing domiciliary oxygen therapy and had previous ICU admissions, showing the severity of disease in this COPD population.

Follow-up studies of ICU patients admitted for general causes are insufficient.^(13,27,28)^ There are few papers on COPD patients with severe exacerbations who require ICU hospitalization^(9,14)^ or that include COPD patients after ICU discharge.^(3,5,8,10)^ Studies evaluating patients with chronic respiratory disease after ICU hospitalization are very important because they add information about the possible benefits of ICU admissions in these chronically ill patients. Therefore, this is an original and relevant study for clinical practice because it allows for a multifaceted view of this population.

The severity of these patient's conditions is likely related to the social reality of our country where most of such patients have never undergone spirometry^(2)^ and are undertreated. In Brazil, patients with COPD are often underdiagnosed, have no access to respiratory physicians and do not receive adequate treatment.^(2)^ The results showed that patients who survived had the potential for improvement in a relatively short period of time. The cause of this improvement may be related to the better management of these COPD patients in a respiratory reference unit.

The data quality was ensured by the selection of patients. To be included in this study, it was necessary to have a clinical diagnosis of COPD prior to or during hospitalization, a history of smoking, and ARF as the cause of ICU admission. In addition, individuals who subsequently had no spirometric criteria for COPD according to GOLD were excluded. The use of objective measures, such as FEV_1_ and 6MWT, and the choice of instruments that widely recognized and validated for use, such as the SGRQ, gives credibility to the results. Careful questionnaire application, in an identical manner, without external interference and by the same trained investigators group, also supports the results.

As reported in a broad population-based study conducted in Sao Paulo,^(2)^ most patients lacked previous pulmonary functional assessment. Only 33% of patients had previously undergone spirometry. As a result, the objective assessment of the disease severity and the adequacy of treatment before ICU admission was not appropriate. COPD was confirmed with later spirometry in all 20 patients who were included in this report. Additionally, there was a significant improvement in the FEV_1_, with an average gain of 290mL in the first month of follow-up, which remained until the end of the three months. After each exacerbation, there is a significant decrease in lung function recorded by the FEV_1_, and there is a subsequent recovery; however, patients usually do not achieve levels comparable to their baseline.^(1,7,8)^ Although patients have shown significant gains in lung function, it is unknown where these individuals are in the trajectory of functional restoration after exacerbation because there is a lack of spirometric data prior to admission.

According to some studies, the mortality of exacerbated COPD patients during the ICU stay is variable.^(3,4,5,7)^ In the present study, the ICU mortality agreed with the APACHE II-predicted mortality (21.6 ± 8.3, 38.9%). Pincelli et al. showed that the mortality rates during the ICU stay and after 28 days of discharge were 20.8% and 33.3%, respectively.^(3)^ Ucgun et al. reported a 33.1% mortality rate in the hospital.^(29)^ In the current study, the mortality rates during the ICU stay, one month after discharge and three months after discharge were 37%, 41% and 44%, respectively.

The relationship between the SGRQ score and mortality has been confirmed, regardless of the severity of bronchial obstruction provided by the FEV_1_.^(11,30)^ A recent systematic review evaluated nine studies on the quality of life after invasive ventilation in COPD and demonstrated that the quality of life deteriorated after invasive ventilation, but the quality of life was similar to patients who are undergoing long-term oxygen therapy or pulmonary rehabilitation programs.^(31,32)^ Rivera-Fernández et al. observed the quality of life of COPD patients after receiving mechanical ventilation at two moments, at discharge and six years later. These authors demonstrated a decrease in the quality of life six years after ICU discharge,^(33)^ which may be due to disease progression during this long post-hospitalization time interval instead of from the impact of the ICU stay. Berkius et al. observed that the health-related quality of life of COPD patients after ICU treatment is lower than in the general population, but 24 months after discharge, the quality of life for these patients was similar to that of COPD patients who were not treated in the ICU.^(34)^ Chiarchiaro et al. showed a decline in the well-being trajectories suddenly after admission to the ICU with recovery in the next 6 months.^(35)^ On the other hand, in the CAOS study, six months after ICU discharge, the majority of survivors considered their quality of life the same as, or better than, before ICU admission;^(5)^ however, the CAOS study included COPD and asthmatic patients. According to Machado et al., patients admitted in the ICU of HU-UFSC for general causes reported a quality of life that was equal to, or better than, after discharge when compared to the quality of life prior to admission. Additionally, there was significant improvement 90 and 180 days after discharge.^(36)^ In accordance with the CAOS study and Machado et al., the present study showed significant improvements in the quality of life. The observed increase in the quality of life for COPD exacerbated patients, detected by the SGRQ in a relatively short period of time, must be highlighted in this study, especially considering the symptom domain.

Although not possible for most patients to complete the 6MWT during the first week after discharge from the ICU, there was significant improvement in performance between the second and third visits. A gain of 54m in the 6MWT after intervention was correlated with clinical improvement.^(24)^ Wise et al. showed a clinically significant difference of 50 to 80m in the 6MWT.^(25)^ In any case, for these patients who have a very low functional capacity, such as the capacity to walk 100m in the 6MWT, a gain of 50m is a significant improvement. Therefore, the best interpretation should be achieved with the difference from the percentage of the basal distance to the distance obtained after intervention, which is considered functional improvement when the gain is more than ten percent of the basal distance value.

Another important finding of this study was a significant improvement in the integrative BODE index during the three-month follow-up period. The increase in the BODE index is related to the increase in hospital admissions and increased number of hospitalization days.^(37)^ Hospital admissions for COPD exacerbation are associated with a higher BODE index.^(38)^ Sanjaume et al. demonstrated that the BODE assessed at discharge predicts mortality in patients who require multiple admissions for COPD exacerbations.^(15)^ Thus far, no study has evaluated the BODE index as a prognostic marker after ICU admission.

Similarly, the cognitive parameters, assessed by the MMSE, showed improvement in the first month after discharge from the ICU and remained stable until the third month of follow-up. Using the MMSE, Ambrosino et al. showed that six months after ICU discharge, the cognition of patients who had already been hospitalized in the ICU for an exacerbation is similar to that in patients who have never previously received intensive care.^(39)^ However, according to Torgersen et al., cognitive deficits can be found in 64% of individuals immediately after the ICU stay, but cognition improves rapidly in the first three months after discharge,^(40)^ as observed in this study.

In the CAOS study, the great majority of COPD and asthma patients who survived the ICU were willing to be readmitted to the ICU, if necessary. Similarly, 80% of patients in the current study also agreed that they might need to return to the ICU. This finding probably indicates that these patients did not experience the ICU stay as a traumatic experience, which may reflect progress in sedation and analgesia and humanization of intensive care. This observation could also have been influenced by the patient perception of improvement in this post-ICU period.

The limitations of this study are the small sample size and relatively short follow-up duration. Nonetheless, we cannot forget that these individuals have extreme difficulty moving themselves. They require family support for outpatient visits and significant physical exertion. Reassessment of these patients in a year or two should be performed to obtain comparative information.

## CONCLUSION

In conclusion, despite the progressive nature of chronic obstructive pulmonary disease and condition severity required for intensive care unit hospitalization, the present study suggests that some patients could have improvement in their clinical, functional and quality of life conditions, even in a brief time interval. Perhaps, the main factor associated with significant clinical improvement after discharge should have been specialist-oriented follow-up, respecting the guidelines to optimize the disease treatment. These findings inspire reflection on the decisions for intensive care unit admission in this population.

## References

[1] Global Strategy for Diagnosis, Management, and Prevention of Chronic
Obstructive Pulmonary Disease.

[2] Nascimento OA, Camelier A, Rosa FW, Menezes AM, Pérez-Padilla R, Jardim JR, Latin American Project for the Investigation of Obstructive Lung
Disease (PLATINO) Group (2007). Chronic obstructive pulmonary disease is underdiagnosed and
undertreated in São Paulo (Brazil): results of the PLATINO
study. Braz J Med Biol Res.

[3] Pincelli MP, Grumann AC, Fernandes C, Cavalheiro AG, Haussen DA, Maia IS (2011). Characteristics of COPD patients admitted to the ICU of a
referral hospital for respiratory diseases in Brazil. J Bras Pneumol.

[4] Ai-Ping C, Lee KH, Lim TK (2005). In-hospital and 5-year mortality of patients treated in the ICU
for acute exacerbation of COPD: a retrospective study. Chest.

[5] Wildman MJ, Sanderson C, Groves J, Reeves BC, Ayres J, Harrison DA (2009). Predicting mortality for patients with exacerbations of COPD and
Asthma in the COPD and Asthma Outcome Study (CAOS). QJM.

[6] Miró Andreu G, Félez Flor M, Solsona Durán JF (2001). Medical decisions in patients with chronic obstructive pulmonary
disease. Med Clin (Barc).

[7] Steer J, Gibson GJ, Bourke SC (2010). Predicting outcomes following hospitalization for acute
exacerbations of COPD. QJM.

[8] Teixeira C, Cabral CR, Hass JS, Oliveira RP, Vargas MA, Freitas AP (2011). Exacerbação aguda da DPOC: mortalidade e estado
funcional dois anos após a alta da UTI. J Bras Pneumol.

[9] Afessa B, Morales IJ, Scanlon PD, Peters SG (2002). Prognostic factors, clinical course, and hospital outcome of
patients with chronic obstructive pulmonary disease admitted to an intensive
care unit for acute respiratory failure. Crit Care Med.

[10] Seneff MG, Wagner DP, Wagner RP, Zimmerman JE, Knaus WA (1995). Hospital and 1-year survival of patients admitted to intensive
care units with acute exacerbation of chronic obstructive pulmonary
disease. JAMA.

[11] Almagro P, Calbo E, Ochoa de Echagüen A, Barreiro B, Quintana S, Heredia JL (2002). Mortality after hospitalization for COPD. Chest.

[12] Wildman MJ, Sanderson CF, Groves J, Reeves BC, Ayres JG, Harrison D (2009). Survival and quality of life for patients with COPD or asthma
admitted to intensive care in a UK multicentre cohort: the COPD and Asthma
Outcome Study (CAOS). Thorax.

[13] Eddleston JM, White P, Guthrie E (2000). Survival, morbidity, and quality of life after discharge from
intensive care. Crit Care Med.

[14] Añón JM, de Lorenzo AG (2009). Prognosis of patients with COPD admitted to the
ICU. Thorax.

[15] Sanjaume M, Almagro P, Rodríguez-Carballeira M, Barreiro B, Heredia JL, Garau J (2009). Post-hospital mortality in patients re-admitted due to COPD.
Utility of BODE index. Rev Clin Esp.

[16] Wildman MJ, O'Dea J, Kostopoulou O, Tindall M, Walia S, Khan Z (2003). Variation in intubation decisions for patients with chronic
obstructive pulmonary disease in one critical care network. QJM.

[17] Menn P, Weber N, Holle R (2010). Health-related quality of life in patients with severe COPD
hospitalized for exacerbations - comparing EQ-5D, SF-12 and
SGRQ. Health Qual Life Outcomes.

[18] Kovelis D, Segretti NO, Probst VS, Lareau SC, Brunetto AF, Pitta F (2008). Validação do Modified Pulmonary Functional Status
and Dyspnea Questionnaire e da escala do Medical Research Council para o uso
em pacientes com doença pulmonar obstrutiva crônica no
Brasil. J Bras Pneumol.

[19] Knaus WA, Draper EA, Wagner DP, Zimmerman JE (1985). APACHE II: a severity of disease classification
system. Crit Care Med.

[20] Miller MR, Hankinson J, Brusasco V, Burgos F, Casaburi R, Coates A, Crapo R, Enright P, van der Grinten CP, Gustafsson P, Jensen R, Johnson DC, MacIntyre N, McKay R, Navajas D, Pedersen OF, Pellegrino R, Viegi G, Wanger J, ATS/ERS Task Force (2005). Standardisation of spirometry. Eur Respir J.

[21] ATS Committee on Proficiency Standards for Clinical Pulmonary
Function Laboratories (2002). ATS statement: guidelines for the six-minute walk
test. Am J Respir Crit Care Med.

[22] Celli BR, Cote CG, Marin JM, Casanova C, Montes de Oca M, Mendez RA (2004). The body-mass index, airflow obstruction, dyspnea, and exercise
capacity index in chronic obstructive pulmonary disease. N Engl J Med.

[23] Folstein MF, Folstein SE, McHugh PR (1975). "Mini-mental state". A practical method for grading the cognitive
state of patients for the clinician. J Psychiatr Res.

[24] Barr JT, Schumacher GE, Freeman S, LeMoine M, Bakst AW, Jones PW (2000). American translation, modification, and validation of the St.
George´s Respiratory Questionnaire. Clin Ther.

[25] Wise RA, Brown CD (2005). Minimal clinically important differences in the six-minute walk
test and the incremental shuttle walking test. COPD.

[26] Schünemann HJ, Griffith L, Jaeschke R, Goldstein R, Stubbing D, Guyatt GH (2003). Evaluation of the minimal important difference for the feeling
thermometer and the St. George`s Respiratory Questionnaire in patients with
chronic airflow obstruction. J Clin Epidemiol.

[27] Stricker KH, Sailer S, Uehlinger DE, Rothen HU, Zuercher Zenklusen RM, Frick S (2011). Quality of life 9 years after an intensive care unit stay: a
long-term outcome study. J Crit Care.

[28] Dowdy DW, Eid MP, Sedrakyan A, Mendez-Tellez PA, Pronovost PJ, Herridge MS (2005). Quality of life in adult survivors of critical illness: a
systematic review of the literature. Intensive Care Med.

[29] Ucgun I, Metintas M, Moral H, Alatas F, Yildirim H, Erginel S (2006). Predictors of hospital outcome and intubation in COPD patients
admitted to the respiratory ICU for acute hypercapnic respiratory
failure. Respir Med.

[30] Oga T, Nishimura K, Tsukino M, Sato S, Hajiro T (2003). Analysis of the factors related to mortality in chronic
obstructive pulmonary disease: role of exercise capacity and health
status. Am J Respir Crit Care Med.

[31] Beer T (2008). What is the health-related quality of life of patients with
chronic obstructive pulmonary disease after invasive
ventilation?. JICS.

[32] Euteneuer S, Windisch W, Suchi S, Köhler D, Jones PW, Schönhofer B (2006). Health-related quality of life in patients with chronic
respiratory failure after long-term mechanical ventilation. Respir Med.

[33] Rivera-Fernández R, Navarrete-Navarro P, Fernández-Mondejar E, Rodriguez-Elvira M, Guerrero-López F, Vázquez-Mata G, Project for the Epidemiological Analysis of Critical Care Patients
(PAEEC) Group (2006). Six-year mortality and quality of life in critically ill patients
with chronic obstructive pulmonary disease. Crit Care Med.

[34] Berkius J, Engerström L, Orwelius L, Nordlund P, Sjöberg F, Fredrikson M (2013). A prospective longitudinal multicentre study of health related
quality of life in ICU survivors with COPD. Crit Care.

[35] Chiarchiaro J, Olsen MK, Steinhauser KE, Tulsky JA (2013). Admission to the intensive care unit and well-being in patients
with advanced chronic illness. Am J Crit Care.

[36] Machado FO, Moritz RD, Margarida CS, Basso G (2007). Avaliação da qualidade e satisfação
de vida dos pacientes antes da internação na Unidade de
Terapia Intensiva e após a alta hospitalar. Rev Bras Ter Intensiva.

[37] Bu XN, Yang T, Thompson MA, Hutchinson AF, Irving LB (2011). Changes in the BODE index, exacerbation duration and
hospitalization in a cohort of COPD patients. Singapore Med J.

[38] Alcázar B, Gaecía-Polo C, Herrejón A, Ruiz LA, de Miguel J, Ros JA (2012). Factors associated with hospital admission for exacerbation of
chronic obstructive pulmonary disease. Arch Bronconeumol.

[39] Ambrosino N, Bruletti G, Scala V, Porta R, Vitacca M (2002). Cognitive and perceived health status in patient with chronic
obstructive pulmonary disease surviving acute on chronic respiratory
failure: a controlled study. Intensive Care Med.

[40] Torgersen J, Hole JF, Kvale R, Wentzel-Larsen, Flaatten H (2011). Cognitive impairments after critical illness. Acta Anaesthesiol Sacand.

